# Investigation of the pressure value while performing biceps tenodesis for superior capsuler reconstruction

**DOI:** 10.1186/s13018-024-04692-1

**Published:** 2024-04-04

**Authors:** Emre Bilgin, Bekir Eray Kilinc, Cigdem Dicle Arican, Baris Yilmaz

**Affiliations:** 1 Department of Orthopedics and Traumatology, Health Sciences University Fatih Sultan Mehmet Training and Research Hospital, D100 Uzeri Hastane Sok. No:1/8 34752 Icerenkoy Atasehir, Istanbul, Turkey; 2 Department of Pathology, Health Sciences University, Prof. Dr. Ilhan Varank Training and Research Hospital, Istanbul, Turkey

**Keywords:** Superior capsular reconstruction, Biceps tenodesis, Massive irreparable rotator cuff tear, Biceps tendon

## Abstract

**Background:**

To compare the histopathological results of biceps tenodesis (BT) performed with normal, low, and high pressures for superior capsule reconstruction (SCR) in rabbits with massive rotator cuff tears.

**Materials and methods:**

Thirty rabbits were divided into three groups. Rabbits 1–10 underwent SCR with BT at the same pressure (Group 1), value measured in the groove; 50% lower (Group 2); 50% higher (Group 3). After the 4-week follow-up, shoulder were en-bloc excised and histopathological evaluation was performed with modified Bonar’s scale. Results were compared between the groups, statistically.

**Results:**

Extracellular matrix were significantly lower in group 2 compared to the other groups (*p* < 0.05). Cellularity levels were significantly lower in group 2 compared to the other groups (*p* < 0.05). Group 2 had no difference between the sides (*p* > 0.05). Group 2 had lower vascularity levels compared to the other groups (*p* = 0.01).

**Dicsussion:**

When the biceps tendon was in the bicipital groove and in a more mobile state with lower pressure exposure. BT performed with a tension that creates less pressure than the biceps in the groove is more successful in SCR.

## Introduction

The surgical treatment of massive irreparable rotator cuff (MIRC) tears continues to be challenging in daily clinical practice. Intramuscular fat infiltration, chronic advanced tendon retraction, and degeneration are the main irreversible factors that contribute to high failure rates in direct repairs [1, 2]. Partial repairs and alternative treatment options, such as rotator interval sliding techniques, have not shown good results in long-term follow-ups, regardless of initial functional improvements [3, 4].

Recent studies have emphasized the physiological importance of the superior capsule of the shoulder joint for the rotator cuff [5]. In relatively young patients with irreparable tears and decreased acromiohumeral distance but without excessive arthritic changes, the reconstruction of the capsule using a fascia lata autograft, called superior capsule reconstruction (SCR), has been described [6]. Successful restoration of glenohumeral stability, increased acromiohumeral distance after surgery, and promising clinical outcomes have been reported with SCR [6, 7].

Several autografts, allografts, xenografts, and synthetic grafts can be used for SCR of the shoulder [8, 9]. Although synthetic grafts have the advantage of causing less donor site morbidity, being easily accessible, and having adjustable dimensions, they result in a significant rise in cost, and there is limited literature for long-term follow-ups on different varieties [10].cFurthermore, allografts have their disadvantages, such as biocompatibility issues and poor functional outcomes after surgery [11]. Therefore, currently, autografts are the most commonly preferred option for SCR. The most frequently used autografts are the biceps tendon and fascia lata graft. The biceps tendon is often the first choice due to its easy accessibility, low cost, and ease of application [12].

There is no existing study on the pressure applied to the biceps tenodesis, a surgical procedure performed for SCR in the treatment of MIRC.

Our study hypothesizes that applying the same pressure to the biceps tendon during biceps tenodesis, which is performed for SCR in the treatment of MIRC, as the pressure measured in the bicipital groove will lead to better healing outcomes.

The aim of our study is to compare the histopathological results after 4 weeks of postoperative follow-up of biceps tenodesis performed with normal, low, and high pressures for SCR in rabbits with massive rotator cuff tears in the same session.

## Materials and methods

This prospective randomized controlled in vivo animal experiment has been approved by the IRB of the authors. Exclusion criteria were determined to exclude animals of different species and ages, those with any diseases, and those previously used in another experiment.

This study was conducted on a total of 30 male New Zealand White rabbits with an average weight of 2 kg and an average age of 12 weeks, totaling 60 shoulders.

### Surgical technique

General anesthesia was induced by 10 mg/kg Xylazine Hydrochloride (Rompun®; Bayer) and 40 mg/kg Ketamine Hydrochloride (Ketalar®; EWL Eczacıbaşı Warner Lambert).

10% Povidone-Iodine (Batticon, Adeka İlaç) was used for antisepsis. A 4-cm skin incision was made at a distance of approximately 4 cm from the bicipital groove. Blunt dissection was performed to locate the biceps tendon.

**Fig. 1:**
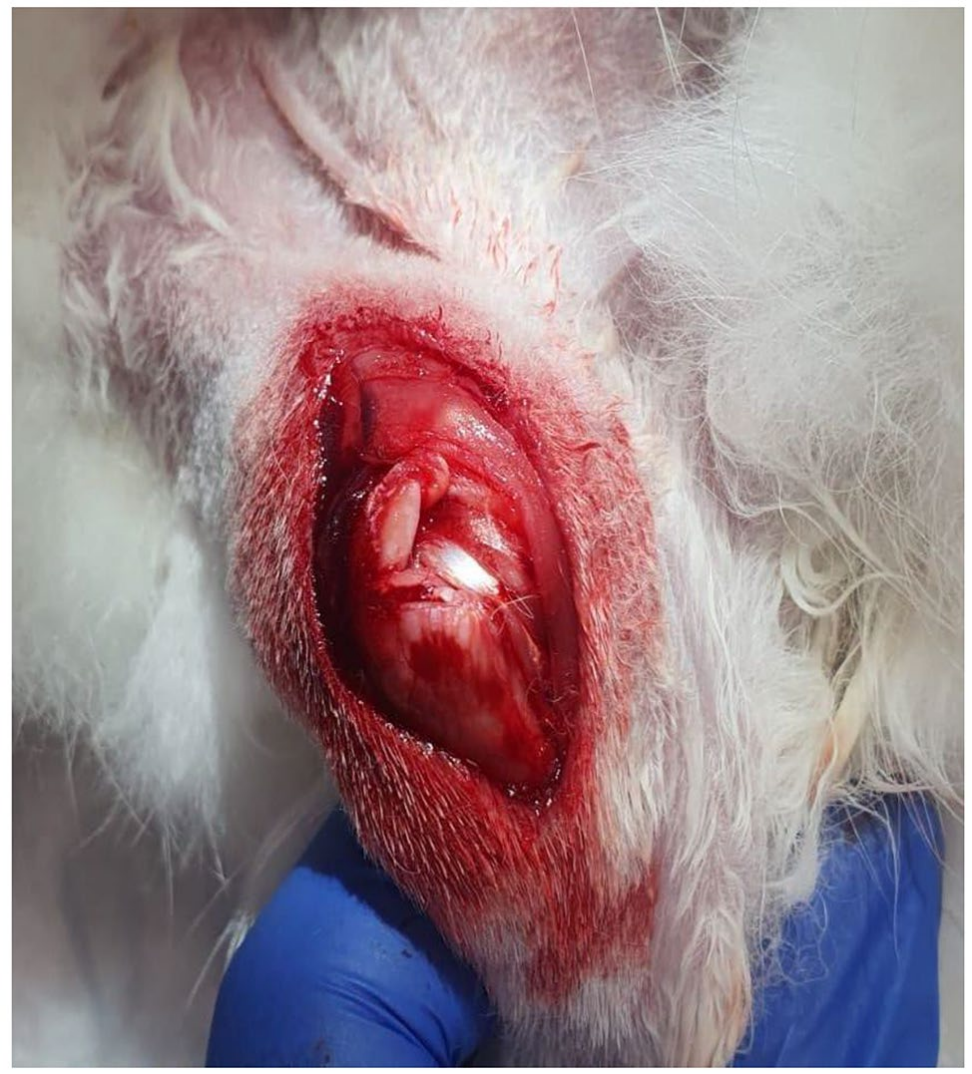
Massive rotator cuff tear

The rabbits were divided into three groups. All animals were subjected to the creation of a massive rotator cuff tear [13] [Fig. 1].

**Fig. 2:**
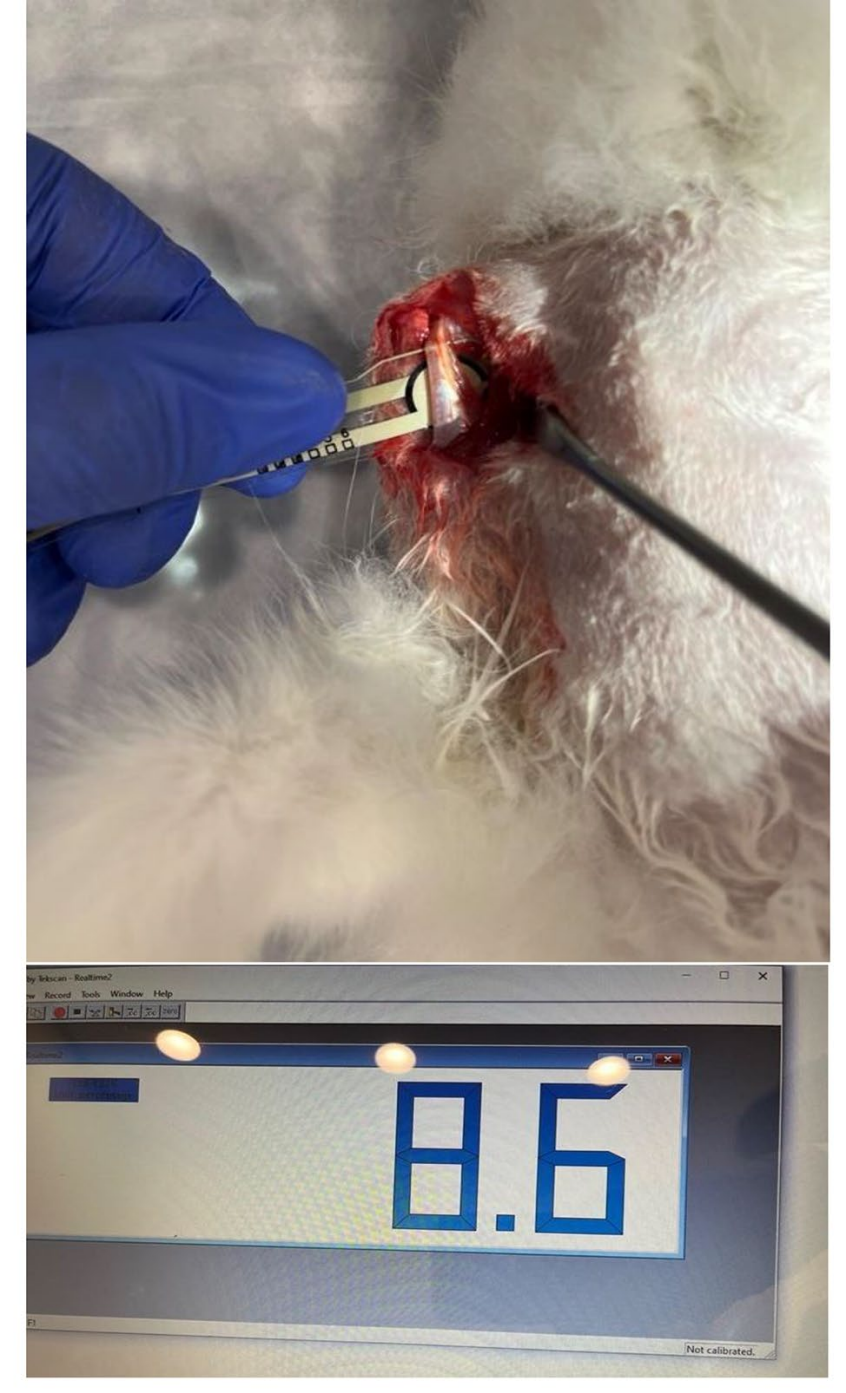
The pressure value between the biceps tendon and the bicipital groove

The pressure value between the biceps tendon and the bicipital groove was measured using the Flexiforce ELF system and B201 sensors (tekscan.com) in a neutral position [Fig. 2].

**Fig. 3:**
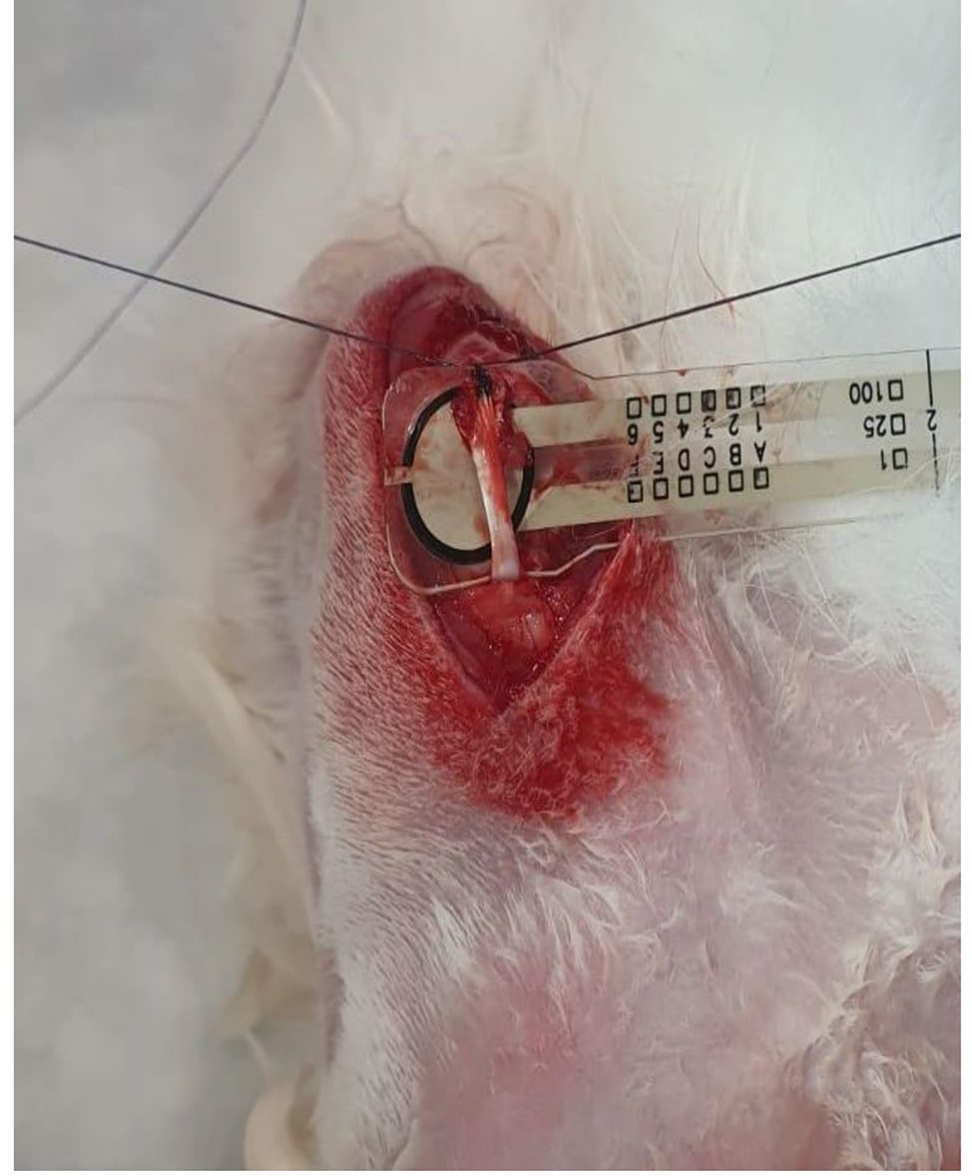
Pressure measurement during BT

#### Group 1

Rabbits 1–10 underwent SCR with biseps tenodesis performed to the footprint using the modified Mason-Allen technique with transosseous sutures (Figure 3) at the same pressure value measured in the groove [Table 1].

**Table 1:** Evaluation between groups

	Groups						*p*
	Group 1		Group 2		Group 3		
	X±s.s.	µ (Min-Max)	X±s.s.	µ (Min-Max)	X±s.s.	µ (Min-Max)	
General Cell Morphology	1.4±1.26	1 (0 -3)	1.15±0.75	1 (0-2)	1±0.67	1 (0-2)	0.80
General Extracellular Matrix	1.45±0.6	1 (1 -2.5)	0.9±0.32	1 (0-1)	1.4±0.52	1 (1-2)	0.04*
General Collagen	1.7±0.48	2 (1 -2)	1.65±0.47	2 (1-2)	1.9±0.32	2 (1-2)	0.35
General Vascularity	1.05±0.69	1 (0 -2)	0.4±0.7	0 (0-2)	0.6±0.52	1 (0-1)	0.04*
General Cellularity	2.00±0.00	2 (2 -2)	1.4±0.7	1.5 (0-2)	1.8±0.42	2 (1-2)	0.03*
General Score (Pathological)	7.65±1.72	7.5 (6-10.5)	5.65±1.93	6 (1-8)	7.2±1.62	7 (4-9.5)	0.04*

The Kruskal-Wallis test was performed

*p<0.05 considered statistically significant

#### Group 2

Rabbits 11–20 underwent biceps tenodesis for SCR at pressure 50% lower than the pressure value measured in the bicipital groove [Table 1].

#### Group 3

Rabbits 21–30 underwent biseps tenodesis for SCR at pressure 50% higher than the pressure value measured in the bicipital groove [Table 1].

Following the procedure, rabbits were administered subcutaneous 0.01–0.05 mg/kg Buprenorfin twice a day until sacrification for analgesia and 30 mg/kg Cefazolin Na ( Cefozin®, Bilim İlaç) twice a day for 3 days. Their mobility was not restricted, and they were kept individually in cages until sacrifice. During the 4-week follow-up period no deterioration in the general health of any rabbit was observed.

**Fig. 4:**
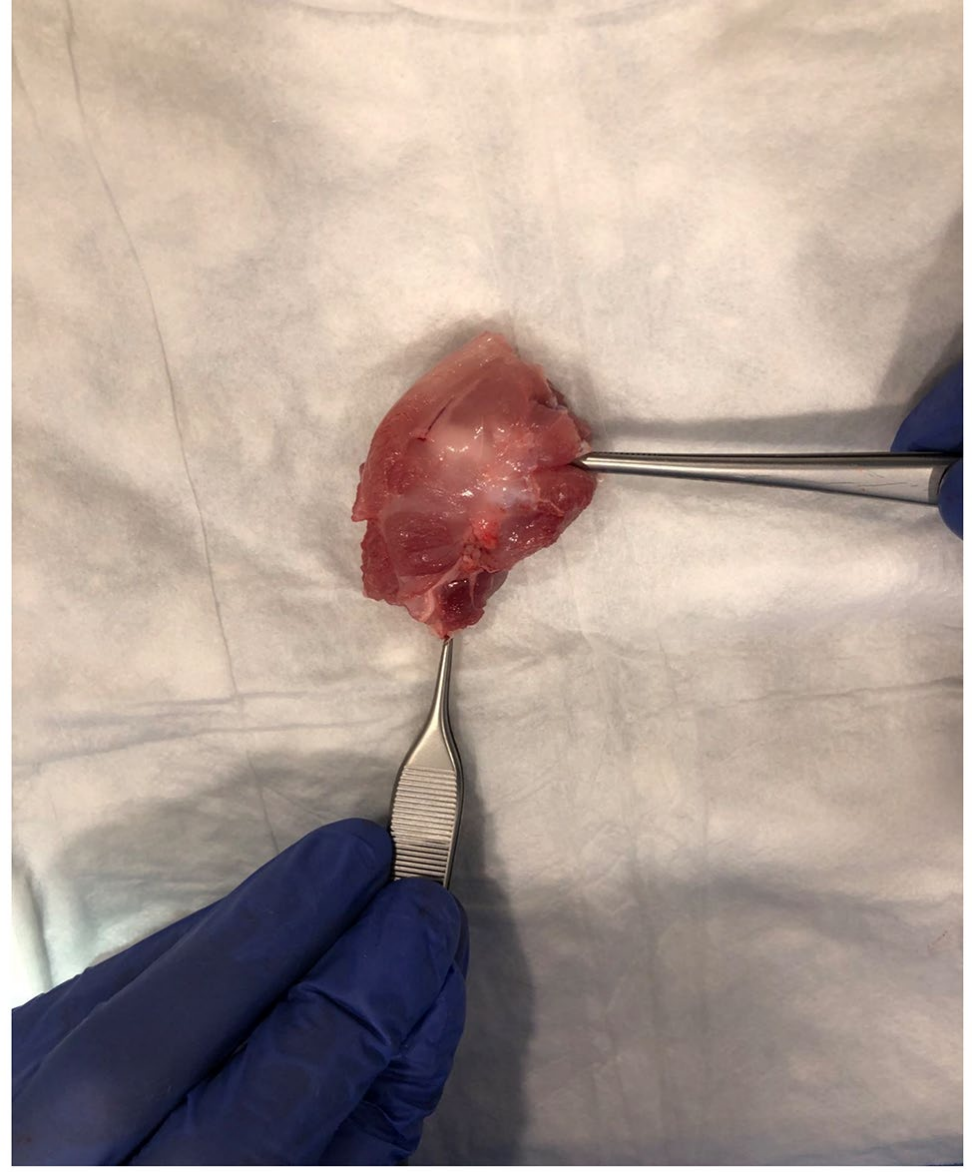
En-bloc excised shoulder joint

After the 4-week follow-up, chemical euthanasia was performed using potassium chloride, and the surgical area, including the scapula and humerus, was en-bloc excised through the same incision [Fig. 4].

### Histopathological evaluation

En-bloc extracted shoulder were fixed in 10% formaldehyde solution for 24 h for tissue preservation.

Subsequently, the tissues were processed in solutions of alcohol, formaldehyde, and xylene for 14 h using a Sakura tissue processor.

The processed tissues were embedded in paraffin blocks using a Thermo Scientific tissue embedding device, and 0.5 mm thick sections were cut on the same brand microtome machine and mounted on slides.

These preparations were stained with Hematoxylin and Eosin and also Masson’s Trichrome staining, which demonstrates muscle and collagen fibers histochemically using the same brand of tissue staining device. Thus, they were prepared for evaluation under a Nikon light microscope.

Cell morphology, extracellular matrix, collagen, vascularity, and cellularity in the tissue were evaluated at magnifications of x100 and x200 using the light microscope.

The modified Bonar’s scale was used for evaluation [14].

### Statistical evaluation

Descriptive statistics were presented as mean ± standard deviation and median-min-max values for continuous variables. The normality of the data was evaluated using the Kolmogorov-Smirnov test. Non-parametric methods were used in the study because the data did not follow a normal distribution (*p* = 0.01), and the sample size was below *n* = 30. The Kruskal-Wallis test was used for comparisons between groups. In the groups that were found to be significant, the Mann-Whitney U test was used to determine the group that differed significantly. The Wilcoxon signed-rank test analysis was used for side-to-side comparisons of measurements within groups. The level of agreement between measurements made by the evaluator at different time points was evaluated using the intraclass correlation coefficient (ICC). A critical decision value of *p* < 0.05 was accepted in the study. The data were analyzed using SPSS 25.0 (Statistical Packages of Social Sciences) software.

## Results

No significant difference was observed in the cell morphology among the groups (*p* > 0.05) [Table 1].

Extracellular matrix were found to be significantly lower in group 2 compared to the other groups (*p* < 0.05) [Table 1].

No significant difference was observed in the collagen among the groups [Table 1].

Vascularity levels were found to be significantly higher in group 1 compared to the other groups (*p* < 0.05) [Table 1].

Cellularity levels were found to be significantly lower in group 2 compared to the other groups (*p* < 0.05) [Table 1].

**Fig. 5:**
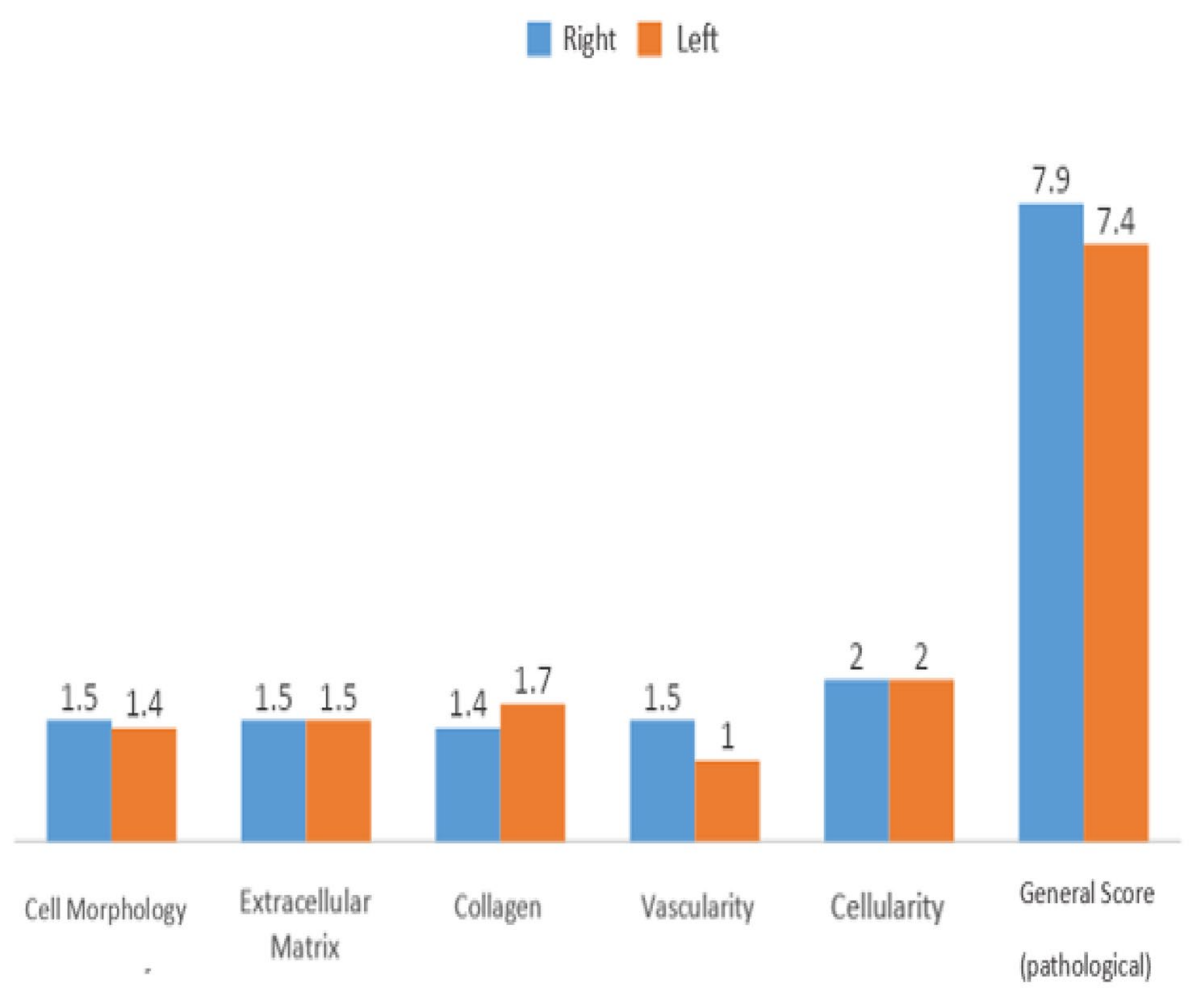
Evaluation of the right and left shoulders for Group 1

In Group 1, no statistically significant difference was observed in the general score evaluation levels between the sides (*p* > 0.05) [Fig. 5].

**Fig. 6:**
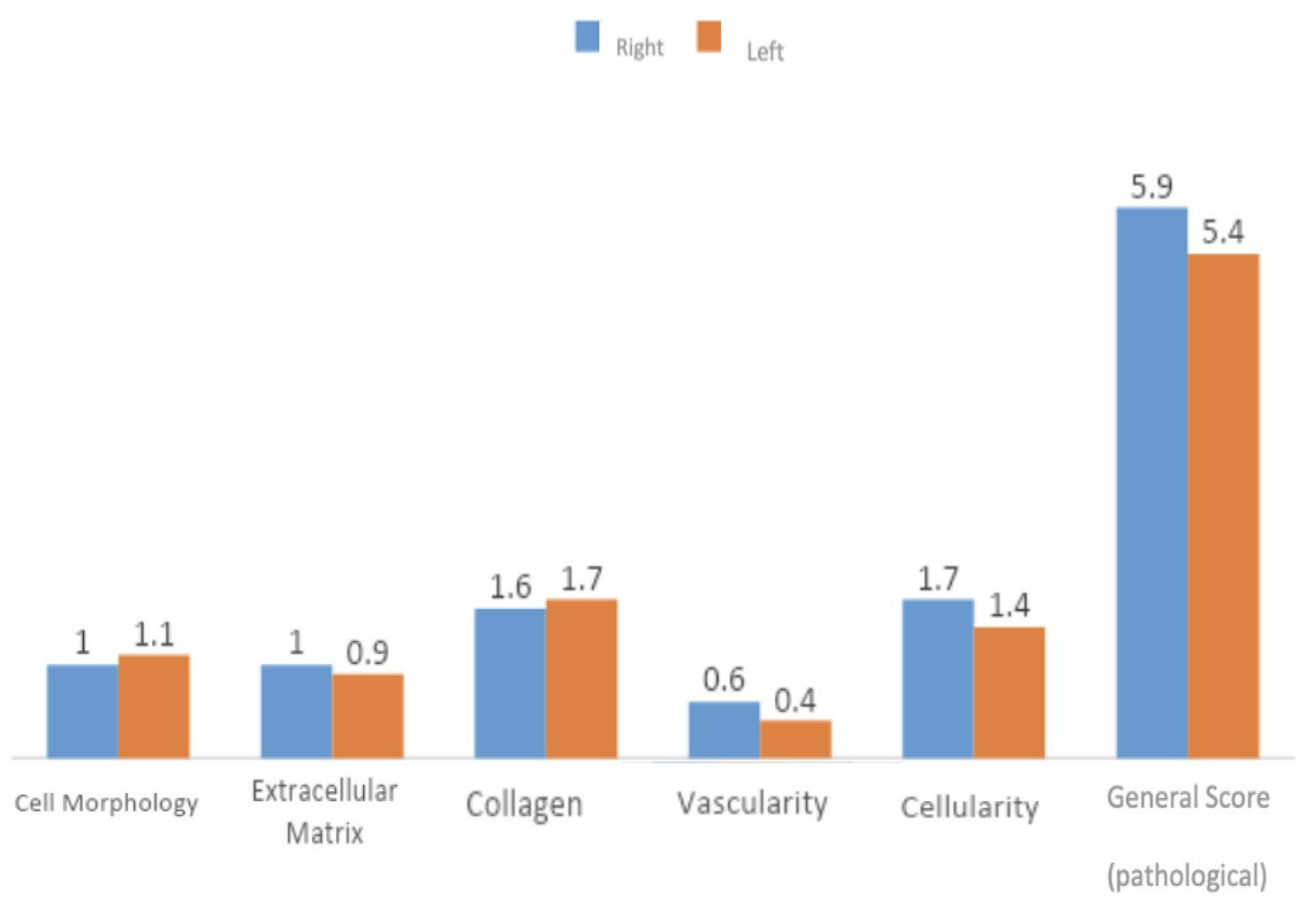
Evaluation of the right and left shoulders for Group 2

In Group 2, no statistically significant difference was observed in the evaluation of general score levels between the sides(*p* > 0.05) [Fig. 6].

**Fig. 7:**
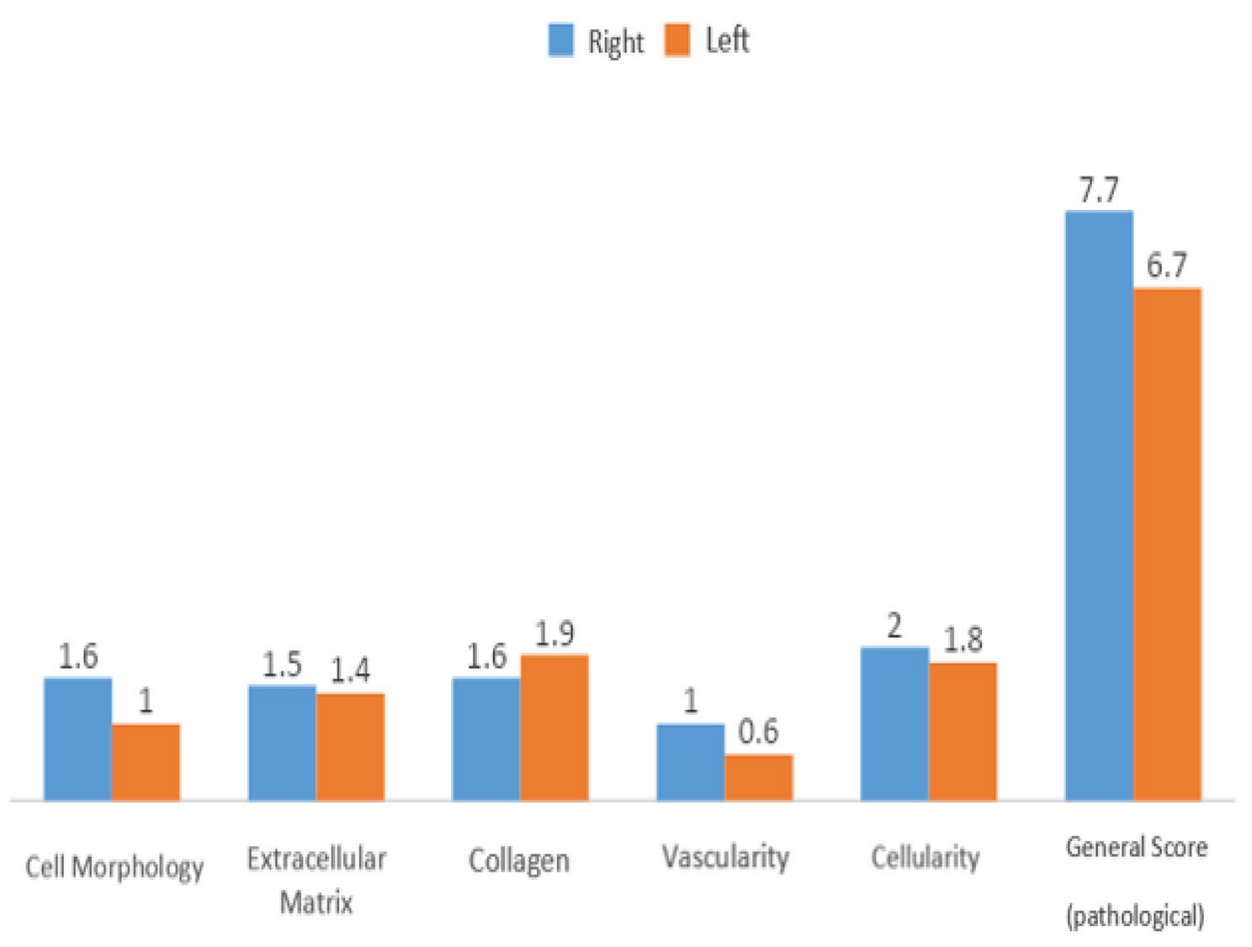
Evaluation of the right and left shoulders for Group 3

In Group 3, no statistically significant difference was observed in the evaluation of general score levels between the right and left shoulders (*p* > 0.05) [Fig. 7].

**Fig. 8:**
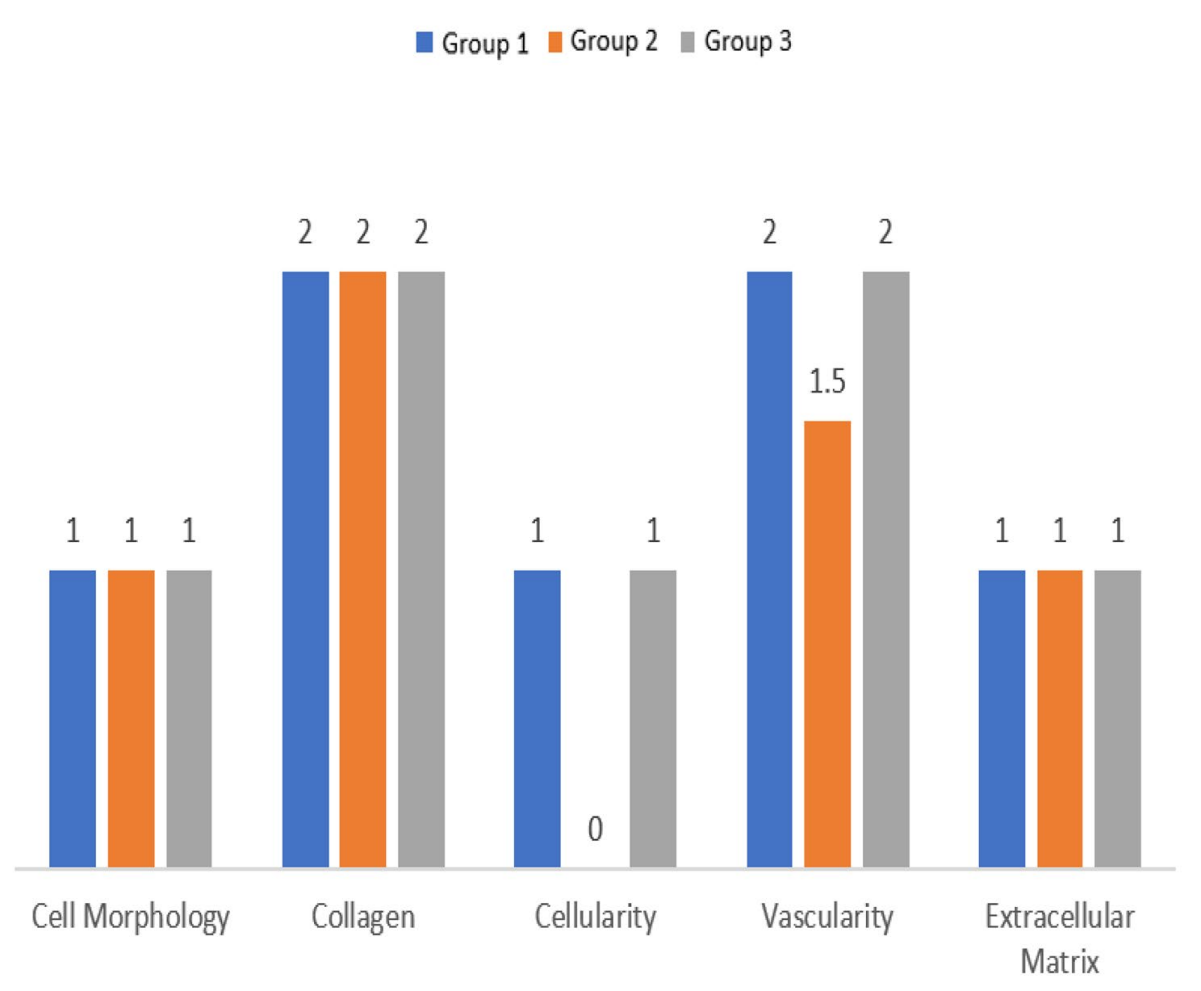
Total Comparisons

No significant differences were found in Cell Morphology measurements between the groups (*p* = 0.34) [Table 2, Figure 8].

**Table 2:** Total Comparisons

	Groups						*p*	Difference
	Group 1		Group 2		Group 3			
	Median	-IQR	Median	-IQR	Median	-IQR		
Cell Morphology	1	(0-3)	1	(1-2)	1	(1-1)	0.34	1-2 *p*>0.05 1-3 *p*>0.05 3-2 *p*>0.05
Collagen	2	(1-2)	2	(1-2)	2	(2-2)	0.41	1-2 *p*>0.05 1-3 *p*>0.05 3-2 *p*>0.05
Cellularity	1	(1-1.5)	0	(0-1)	1	(0-1)	0.01*	1-2 *p*=0.01*
								1-3 *p*>0.05
								3-2 *p*=0.01*
Vascularity	2	(2-2)	1,5	(1-2)	2	(2-2)	0.02*	1-2 *p*=0.01*
								1-3 *p*>0.05
								3-2 *p*=0.01*
Extracellular Matrix	1	(1-2)	1	(1-1)	1	(1-2)	0.56	1-2 *p*>0.05 1-3 *p*>0.05 3-2 *p*>0.05

Kruskal-Wallis test and Mann-Whitney U test was used to analyze the difference

*p<0.05 considered statistically significant

No significant differences were found in Collagen measurements between the groups (*p* = 0.41) [Table 2, Figure 8].

No significant differences were found in Extracellular Matrix measurements between the groups (*p* = 0.56) [Table 2, Figure 8].

Group 2 had lower Vascularity levels compared to the other groups (*p* = 0.01) [Table 2, Figure 8].

## Discussion

Contrast to our hypothesis, we found that the biceps tendon’s lower pressure during this tenodesis procedure was more successful in terms of healing compared to being under normal and high pressure.

The incidence of massive rotator cuff tears goes up with advancing age [7]. Especially in cases where treatment is delayed, severe muscle retraction and fatty degeneration of the muscles are observed. These factors have been described as irreparable tears due to the significantly increased risk of re-tear after repair [6]. Arthroscopic SCR and arthroscopic latissimus dorsi transfer (ALDT) are some alternative surgical procedure for this condition. Osti et al. reported that ALDT had shown a greater complication rate and less improvement in acromio-humeral distance [15]. However, research is still ongoing to determine the optimal graft for SCR and the optimal technique for graft application [8, 16]. A study reported that both graft preferences either autograft or synthetic allograft transplantation had satisfactory results [17]. In our study, we investigated the pressure level that the biceps tendon should have during tenodesis applied to the footprint of the rotator cuff while preserving the anchor of the biceps tendon in the superior glenoid for SCR. Since the effect of pressure values during biceps tenodesis on healing has not been previously investigated, our study will contribute to the literature and shed light on future studies.

Studies have shown successful results of biceps augmentation procedures in addition to rotator cuff repair in chronic rotator cuff tears [18]. In a study where biceps augmentation surgery was performed, patients experienced higher functional outcomes and rapid reduction in pain compared to the control group that underwent repair alone [19]. Another study used the biceps tendon as a free graft by tenotomizing it to avoid high tension during repair and demonstrated significant improvement in patient’s clinical scores and faster recovery of muscle strength in postoperative follow-ups [20]. Biceps tendon is preferred as an autograft in previous studies are the absence of the need for additional incisions, low cost, and no donor site morbidity [21].

Due to the numerous advantages over other graft types, we believe that the long head of the biceps tendon will continue to be frequently used for SCR. Therefore, we conducted our study using the biceps tendon to shed light on future studies. In our surgical technique, the biceps tendon functions as a dynamic stabilizer of the shoulder joint with mobility and lower pressure exposure in its natural localization in the bicipital groove. After tenodesis of the biceps tendon for SCR, it assumes a static stabilizer role and is subjected to a higher pressure than its natural state. We also believe that it cannot show flexibility against pressure due to limited mobilization. As a result, we found histological evidence of better healing with a lower pressure value measured in the groove compared to SCR performed with normal pressure or higher pressure.

A study demonstrated that SCR with augmentation of the biceps autograft improved glenohumeral stability and reduced subacromial peak contact pressure in irreparable supraspinatus tears [21]. A new technique was proposed to perform rotator cuff repair with biceps augmentation by creating a new bicipital groove without fixing the biceps tendon, allowing it to progress in the newly created groove for the SCR [22].

Particularly, there is a lack of studies on the level of pressure that should be applied by the graft to the repair area during the procedure. We observed a lack of consensus regarding the optimal application technique of the commonly used biceps tendon for SCR and the optimal pressure applied during the grafting procedure. In our technique we tenodesed the distal part of the biceps tendon to the footprint of the rotator cuff. One of the greatest advantage of this technique is that the proximal part of the biceps tendon is already naturally anchored to the superior glenoid.

In cases where the biceps tendon was used, greater improvement in acromiohumeral distance was observed, but there was no difference in the incidence of postoperative re-tear [23]. Due to better functional outcomes, it was recommended to perform SCR with the biceps tendon rather than using a fascia lata graft [8]. Most studies have highlighted the use of the biceps tendon as being more minimally invasive compared to the use of fascia lata graft, with no donor site morbidity. Follow-ups after surgical treatment have shown advantages in terms of clinical and radiological parameters compared to the use of fascia lata grafts [8, 23]. One of the main reasons for our preference for the biceps tendon in our study for SCR is its benefits over other autografts in the treatment of irreparable massive rotator cuff tears, and we believe it will be more commonly preferred in future research.

A study recommended that the technique of creating a patch from the biceps tendon, which will be used as an autograft, by meshing it with a meshing device, due to its high healing potential, low cost, and absence of donor site morbidity [24].

In our daily practice, a specific technique that has proven superiority over other techniques for SCR has not yet been established. In our study, we preferred the Chinese method technique, which describes the suture anchor fixation of the rotator cuff to the footprint after biceps tenotomy, due to its ease of application and the absence of the need for an additional incision [2]. In an acute massive rotator cuff tear created in a rabbit model, we demonstrated through histopathological evaluation that tenodesis the biceps tendon with lower pressure compared to normal significantly increased the success rate of biceps tenodesis for SCR, using a device called Flexiforce for pressure measurement. When the biceps tendon was in the bicipital groove and in a more mobile state with lower pressure exposure, the healing rates of tenodesis performed with normal or high pressure were significantly lower compared to those performed with low pressure, as the biceps tendon was subjected to higher pressure as a static stabilizer after the procedure. We assume that the pressure exerted by the graft during biceps tenodesis is important for the successful completion of the SCR procedure. We consider pressure measurement valuable to ensure proper graft healing and prevent complications.

Despite these findings, we have some limitations in our study. Firstly, since our study was conducted on an experimental animal population, there are biological differences between humans and these animals. Some of the differences include their shoulder joints being weight-bearing joints, having a high healing potential, and having relatively different shoulder anatomy. Therefore, these factors may affect our histopathological evaluation. And we also couldn’t use immunohistochemical evaluation for this study. Yet, we used the modified Bonar’s scale with all parameters for the evaluation of the healing. Additionally, since we applied an acute injury model in our study, the recovery of repairs applied in the treatment of rotator cuff tears caused by chronic degenerative pathologies in humans may not be the same. Furthermore, the use of the same species and breed in our study, along with the homogeneous distribution of groups and the prospective design, are strengths of our study.

## Conclusion

In our study conducted on rabbits, we conclude that biceps tenodesis performed with a tension that creates less pressure than the biceps in the groove is more successful in SCR aimed at repairing irreparable massive rotator cuff tears. Based on this information, this animal study to determine the outcome of our biceps tenodesis will serve as a reference for future clinical studies in our approach to treating irreparable rotator cuff tears.

## Data Availability

No datasets were generated or analysed during the current study.
